# Adiponectin reduces carotid atherosclerotic plaque formation in ApoE^−/−^ mice: Roles of oxidative and nitrosative stress and inducible nitric oxide synthase

**DOI:** 10.3892/mmr.2014.2947

**Published:** 2014-11-13

**Authors:** XIAOJUN CAI, XUAN LI, LI LI, XIAO-ZHEN HUANG, YU-SHENG LIU, LIANG CHEN, KE ZHANG, LIN WANG, XIAONAN LI, JIANTAO SONG, SHUZHEN LI, YUN ZHANG, MEI ZHANG

**Affiliations:** 1 The Key Laboratory of Cardiovascular Remodeling and Function Research, Chinese Ministry of Education and Chinese Ministry of Public Health, Shandong University Qilu Hospital, Jinan, Shandong 250012, P.R. China; 2 Department of Cardiology, Jinan Central Hospital Affiliated to Shandong University, Shandong 250014, P.R. China; 3 Department of Cardiology, Weifang Traditional Chinese Medicine Hospital, Weifang, Shandong 261041, P.R. China; 4 Department of Cardiology, Jinan Huaiyin Hospital, Shandong 250021, P.R. China; 5 Department of Cardiology, The Second Hospital of Shandong University, Shandong 250033, P.R. China

**Keywords:** adiponectin, atherosclerosis, oxidative stress, nitrite/nitrate, inducible nitric oxide synthase

## Abstract

Adiponectin (APN) is an important anti-atherogenic adipocytokine. The aim of the present study was to investigate the role of adiponectin in atherosclerotic plaque formation and clarify its mechanisms. An atherosclerosis model was induced by *in vivo* perivascular constrictive silica collar placement on the left common carotid arteries in male apolipoprotein E-deficient (ApoE^−/−^) mice. All of the mice were fed a high-fat diet, and divided into phosphate-buffered saline, adenovirus (Ad)-β-galactosidase and Ad-APN treatment groups. Compared with treatment of Ad-β-gal or PBS, Ad-APN treatment markedly reduced inducible nitric oxide synthase (iNOS) protein expression, decreased in nitric oxide/superoxide production, blocked peroxynitrite formation and reversed the progression of atherosclerotic lesions. Adiponectin may be a natural molecule that reduces atherosclerosis by inhibiting iNOS and consequently diminishing oxidative/nitrative stress.

## Introduction

All of the clinical and experimental studies on adiponectin (APN) markedly suggest that it is a critical vascular protective molecule and its reduction may contribute to vascular injury in metabolic disorder-associated diseases (1–5). Adiponectin is an adipose-specific plasma protein that has important roles in atherosclerosis, obesity and type 2 diabetes (6–8). Experimental studies have demonstrated that adiponectin has anti-inflammatory and anti-atherogenic properties. Previous studies have demonstrated that adiponectin overexpression reversed abnormal neointimal thickening in adiponectin-deficient mice and alleviated atherosclerotic lesions in apolipoprotein E-deficient (ApoE^−/−^) mice. Okamoto *et al* (9) reported that injection of an adenovirus containing adiponectin into ApoE^−/−^ mice significantly reduced lesions within the aortic sinus region, and the results suggested that adiponectin may mediate its anti-atherogenic effects by adhering to macrophages and actin-negative cells (endothelial cells) within the fatty streak lesions. The study also revealed that adiponectin decreased the size of lipid droplets, vascular cell adhesion protein 1 expression and class A scavenger receptor without affecting lipid profiles in the plasma (9).

Oxidative stress, comprising the production of ·O_2_^−^ and its derivative peroxynitrite (ONOO^−^), contributes to the onset of atherosclerosis (10). The levels of circulating adiponectin are inversely correlated with the plasma levels of oxidized low-density lipoprotein (LDL) in patients with type 2 diabetes and coronary artery disease, which suggests that low adiponectin levels are associated with an increased oxidative state in the arterial wall (11). Reduced adiponectin levels have also been correlated with increased levels of systemic oxidative stress (12). Furthermore, elevated endothelial reactive oxygen species (ROS) production stimulated by multiple agonists, including oxidized adiponectin and high glucose levels, was dose-dependently suppressed *in vitro* by recombinant globular adiponectin (13–14). Adiponectin improved endothelial function in hyperlipidemic rats (15) and protected hearts against ischemia/reperfusion injury by reducing oxidative/nitrative stress (16). Therefore, adiponectin may be a potent anti-oxidative/nitrative stress agent. However, to the best of our knowledge, whether adiponectin overexpression reduces atherosclerotic plaque by acting on oxidative/nitrative stress has not been investigated to date.

Therefore, the present study aimed to determine the relevance of adiponectin and its attenuation of oxidative stress. It was identified that adiponectin may reduce atherosclerotic plaque and improve the stability of plaques in ApoE^−/−^ mice with atherosclerosis by inhibiting inducible nitric oxide synthase (iNOS) and oxidative/nitrative stress.

## Materials and methods

### Animals

The adenovirus producing the full-length adiponectin was constructed with use of the Adenovirus (Ad) Expression Vector kit (Takara, Kyoto, Japan) as described previously (17). From the effects of different titers of Ad-APN on serum adiponectin concentration in mice by ELISA, the optimal concentration of Ad-APN of 2×10^8^ plaque-forming units [pfu] of infection was used.

Male ApoE^−/−^ mice (10–12 weeks old; Beijing University, Beijing, China) were housed at room temperature (24°C) under a 12-h dark/light cycle and received a high-fat diet (0.25% cholesterol and 15% cocoa butter) for 12 weeks. Carotid atherosclerotic lesions were induced by surgical placement of perivascular constrictive silica collars (Shandog Medical Instrument Institute, Shandong, China) on the left common carotid arteries as described by our group previously (17). Eight weeks following surgery, the mice were divided into three groups for treatment (n=30/group): Phosphate-buffered saline (PBS), Ad-β-galactosidase (Ad-β-gal) and Ad-APN groups. The silica collars on the left common carotid arteries were removed carefully. PBS, Ad-β-gal and Ad-APN were trickled into the silica collar parts of common carotid arteries of mice. Two weeks following treatment, the mice were sacrificed and carotids were excised. The experiment conformed with the Guide for the Care and Use of Laboratory Animals published by the US National Institutes of Health (NIH Publication no. 85–23, revised 1985) and Shandong University guidelines (Shandong, China). This study was approved by the ethics committee of Jinan Central Hospital affiliated to Shandong University (Jinan, China).

### Micro-ultrasonography

The Vevo770 ultrasonography system (Visualsonics, Toronto, Canada; 55-MHz scan head, 4.5-mm focus, axial resolution 30 μM) was used to measure the baseline parameters of the left carotid artery at the beginning of the experiment prior to and following transfection as previously described (18).

### Histology

Frozen carotid-artery cross-sections (10-μM thick) embedded in optimal cutting temperature compound (OCT; Sakura Finetechnical Co., Ltd., Tokyo, Japan) following overnight fixation in 10% formalin were mounted on slides. Three sections (200-μM apart) for each mouse were stained with Oil-red O (Ameresco, Solon, OH, USA) and Masson trichrome (Maxim-Bio, Fujian, China). The corresponding sections were measured by use of an automated image analysis system (Image-Pro Plus 5.0, Media Cybernetics, Rockville, MD, USA) attached to a color CCD video camera (BX51; Olympus Corp., Tokyo, Japan).

### Determination of total nitric oxide (NO) in carotid tissue

The carotid tissue was rinsed and homogenized. The content of NO in the supernatant was determined using the Griess Reagent method (Nitric Oxide Assay kit, Beyotime, Shanghai; Siever 280i NO Analyzer) as described previously (17).

### Quantification of superoxide production

*In situ* superoxide content was detected by dihydroethidium staining (DHE; Molecular Probes, Carlsbad, CA) as described previously(19). The carotid tissue superoxide content was determined by lucigenin-enhanced luminescence (20).

### Immunoblotting

The protein from the carotid tissue homogenates was separated by SDS-PAGE, transferred to nitrocellulose membranes and incubated with monoclonal antibodies against adiponectin, iNOS (both Upstate Biotechnology, Inc., Lake Placid, NY, USA) and β-actin (Cell Signaling Technology, Inc., Beverly, MA, USA), then horseradish peroxidase-conjugated anti-mouse immunoglobulin G antibody (1:2,000, Cell Signaling Technology, Inc., Danvers, MA, USA) for 1 h. The blots were developed by using a supersignal chemiluminescence kit (Pierce Biotechnology, Inc., Rockford, IL, USA) and visualized using a Kodak Image Station 400 (Amersham Pharmacia, Deisenhofen, Germany). The sample loadings were normalized with anti-β-actin polyclonal antibody (Sigma-Aldrich, St. Louis, MO, USA) and quantification involved use of the Image Station 2000R system (Eastman Kodak, Rochester, NY, USA).

### Immunohistochemistry

Paraformaldehyde-fixed tissues were cut into semi-thin sections 4–5-μM thick and stained with the following antibodies: mouse anti-macrophage/monocyte monoclonal (clone MOMA-2; 1:500; Millipore, Billerica, MA, USA), mouse anti-human anti-α-actin (1:500; Millipore) and mouse anti-nitrotyrosine (clone 1A6; 1:200; Millipore). Immunohistochemistry staining was developed using a Vectastain ABC kit (Vector Laboratories, Burlingame, CA, USA).

### Determination of plasma APN concentration

Plasma adiponectin levels were determined by use of a mouse adiponectin ELISA kit (Phoenix Pharmaceuticals, Inc., Belmont, CA, USA) according to the manufacturer’s instructions.

### Quantification of tissue nitrotyrosine content

The nitrotyrosine content in the carotid tissue, indicating *in vivo* ONOO^−^ formation, an index of nitrative stress, was determined by use of a nitrotyrosine ELISA kit (Cell Sciences, Canton, MA, USA) according to the manufacturer’s instructions.

### Statistical analysis

All data are expressed as the mean ± standard error. Comparisons between the various groups were analyzed by one-way analysis of variance followed by Dunnett’s post-hoc test using SPSS 16.0 software (International Business Machines, Armonk, NY, USA). P<0.05 (two-sided) was considered to indicate a statistically significant difference.

## Results

### Ad-APN transfection increased APN levels in carotid arteries of ApoE^−/−^ mice

**Figure 1:**
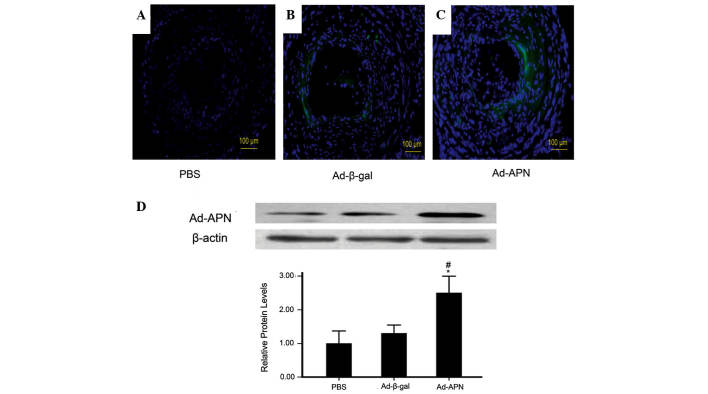
APN levels in atherogenic plaque in ApoE^−/−^ mice. Green fluorescence protein expression in atherogenic plaque seven days following injection with (A) PBS, (B) Ad-β-gal and (C) Ad-APN (magnification, ×10). (D) Relative APN levels in atherogenic plaque in PBS, Ad-β-gal and Ad-APN groups. Values are expressed as the mean ± standard deviation. ^*^P<0.05 vs. control; ^#^P<0.05 vs. Ad-β-gal. PBS, treated with PBS; Ad-β-gal, transfected with empty vector; Ad-APN, transfection with Ad-APN. ApoE^−/−^, apolipoprotein E-deficient; Ad-β-gal, Ad-β-galactosidase; PBS, phosphate-buffered saline; APN, adiponectin; Ad, adenovirus.

On the 7th day following virus injection, the expression of virus-targeted genes was detectable in the mice, without loss of weight. As demonstrated in Fig. 1A–C, ~50% GFP expression in the Ad-APN plaques and 52% in the Ad-β-gal plaques but not in PBS plaques indicated effective transfection.

Protein levels of adiponectin in the carotid tissue were further studied. The results demonstrated that protein levels of adiponectin in Ad-APN plaques were higher than those in the Ad-β-gal and PBS plaques (2.5±0.2 vs. 1.3±0.1; P<0.05; 2.5±0.2 vs. 1.0±0.15; P<0.05; Fig. 1D). There was no significant difference between the control groups (1.0±0.15 vs. 1.3±0.1; P>0.05; Fig. 1D). Therefore, Ad-APN transfection efficiently increased the levels of adiponectin in mouse carotid arteries.

### APN reduces atherosclerotic lesions in carotid arteries in ApoE^−/−^ mice

**Figure 2:**
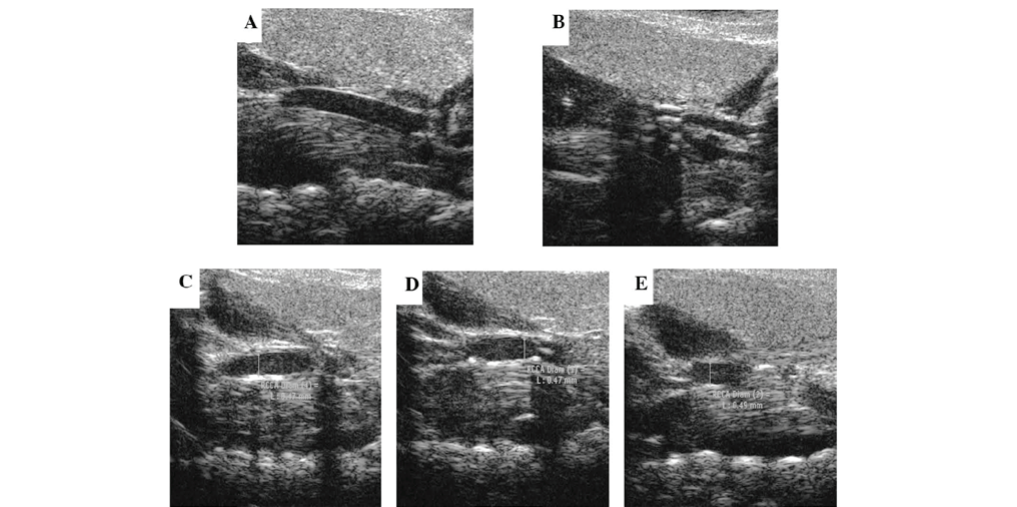
Micro-ultrasonographic evaluation of the plaque. (A) The carotid intima of control mice was smooth prior to surgery. (B) Abundant atherosclerotic lesions were visible in the lumen six weeks following surgery. (C–E) Following two weeks of transfection, the atherosclerotic plaques in the left common carotid arteries were observed on micro-ultrasonography in (C) the PBS group, (D) The Ad-β-gal group and (E) the Ad-APN group. PBS, treated with PBS; Ad-β-gal, transfected with empty vector; Ad-APN, transfected with adiponectin adenovirus. Ad-β-gal, Ad-β-galactosidase; PBS, phosphate-buffered saline.

The present study used micro-ultrasonography to evaluate the plaques. Prior to surgery, the carotid intima of control mice was smooth (Fig. 2A). Six weeks following surgery, abundant atherosclerotic lesions were present in the lumen (Fig. 2B). The carotids were also compared following collar removal and 14 days of PBS treatment to demonstrate that this remained the same (Fig. 2B and C). The results revealed that the removal of the collar did not improve atherosclerosis itself, which was improved by Ad-APN only. Following two weeks of transfection, the lesion sizes in the APN group were significantly lower than those in the Ad-β-gal and PBS groups, while there was no significant difference between the control groups (Fig. 2C–E).

**Figure 3:**
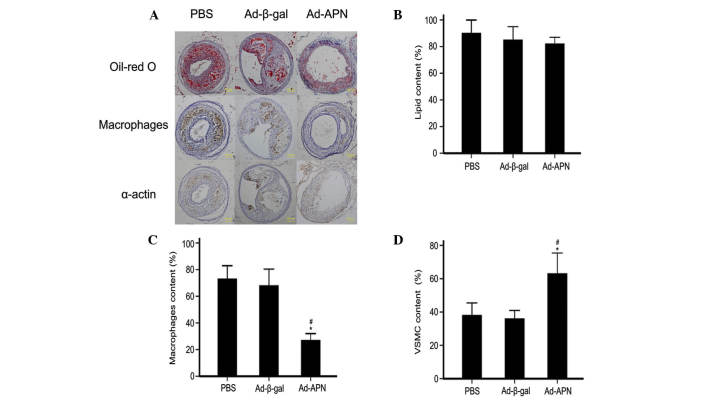
Effect of APN on plaque composition in carotid atherosclerotic lesions in ApoE^−/−^ mice. (A) Representative microscopic images of the types of atherosclerotic plaques (magnification, ×20). The plaque composition was comparable among treatment groups (PBS, Ad-β-gal transfection and Ad-APN transfection) two weeks following transfection. (B–D) Quantification of the immunohistochemical stains. (B) Lipid content assessed by Oil-red O staining; (C) immunohistochemical detection of macrophages; and (D) α-actin, for smooth muscle in atherosclerotic lesions. PBS, transfection with PBS; Ad-β-gal, transfection with empty vector; Ad-APN, transfection with Ad-APN. ApoE^−/−^, apolipoprotein E-deficient; PBS, phosphate-buffered saline; APN, adiponectin; Ad, adenovirus; VSMC, vascular smooth muscle cell.

Based on the micro-ultrasonography results, the plaque composition was further analyzed (Fig. 3A). Transfection with Ad-APN did not decrease the lipid contents, as there was no significant difference between any of the groups (82 vs. 86%, P>0.05; 82% vs. 90%, P>0.05; 86 vs. 90%, P>0.05; Fig. 3B). Ad-APN transfection produced a higher macrophage content than Ad-β-gal transfection (73 vs. 68%; P<0.05), with no difference between the control groups (68 vs. 0.27%; P>0.05; Fig. 3C). Transfection with Ad-APN produced a greater vascular smooth muscle cell (VSMC) content than transfection with Ad-β-gal (63 vs. 36%; P<0.05), while there was no significant difference between the control groups (36 vs. 38%, P>0.05; Fig. 3D).

### APN decreases superoxide, NO and ONOO^−^ production in ApoE^−/−^ mice

**Figure 4:**
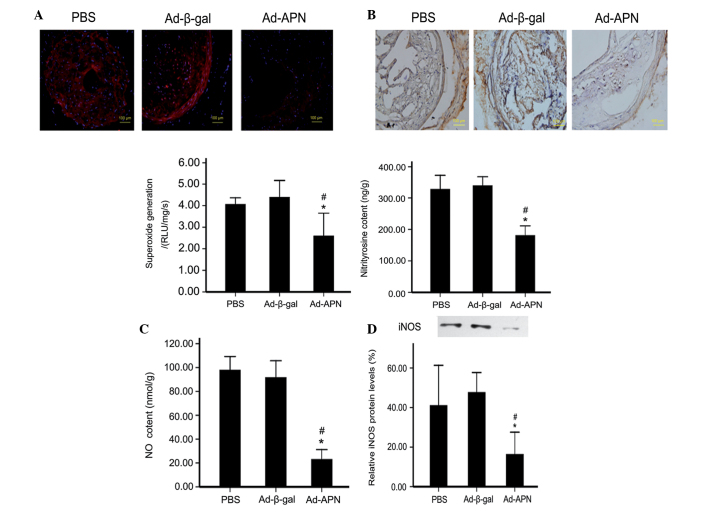
Effect of APN on oxidative/nitrative stress in carotid atherosclerotic lesions in ApoE^−/−^ mice. (A) Detection of superoxide content (magnification, ×40). (B) Immunohistochemical detection of nitrotyrosine and nitrotyrosine content in carotid tissues (magnification, ×40). (C) NO content. (D) Western blot analysis of iNOS protein levels. The values are the mean ± standard deviation. ^*^P<0.05 vs. control; ^#^P<0.05 vs. Ad-β-gal. PBS, treated with PBS; Ad-β-gal, transfected with empty vector; Ad-APN, transfected with Ad-APN. iNOS, inducible nitric oxide synthase; ApoE^−/−^, apolipoprotein E-deficient; PBS, phosphate-buffered saline; APN, adiponectin; Ad, adenovirus.

Oxidative and nitrative stresses are primary factors responsible for atherosclerosis. To further assess the mechanism of altered atherosclerotic lesions in ApoE^−/−^ mice, superoxide and NO production were detected. As summarized in Fig. 4, Ad-APN transfection reduced the enhanced superoxide production in the carotid tissue of ApoE^−/−^ mice, compared with that in the Ad-β-gal-transfected mice (2.47.1±0.9 vs. 4.43±0.6 RLU/mg/s); P<0.05; Fig. 4A), while there was no difference between the control groups (4.43±0.6 vs. 4.10±0.2 RLU/mg/s; P>0.05; Fig. 4A). In addition, NO production was decreased in the Ad-APN-treated mice as compared with the Ad-β-gal-treated mice (22±3 vs. 96±5 nmol/g; P<0.05; Fig. 4B), with no difference between the control groups (96±5 vs. 97±4 nmol/g; P>0.05; Fig. 4B). Therefore, adiponectin reduced atherosclerosis in ApoE^−/−^ mice, possibly through reducing superoxide production and preventing NO destruction. In addition, the content of ONOO^−^, the biradical reaction product of superoxide and NO, was detected by measurement of nitrotyrosine, a marker of *in vivo* ONOO^−^ production. It was identified that the nitrotyrosine content was reduced in the Ad-APN-treated mice compared with that in the Ad-β-gal-treated mice (189±12 vs. 331±13 ng/g; P<0.05; Fig. 4C), with no difference between the control groups (331±13 vs. 325±25 ng/g; P>0.05; Fig. 4C).

### Treatment with adiponectin decreases NO production by downregulating iNOS expression in ApoE^−/−^ mice

The aforementioned results suggested that the reduced superoxide production and prevention of NO destruction may contribute to the vasculoprotective effect of APN in ApoE^−/−^ mice. To identify the molecular candidates responsible for this effect, the iNOS protein expression was determined. It was identified that iNOS activity was reduced in the Ad-APN-treated mice (P<0.05; Fig. 4D) compared with the Ad-β-gal-treated mice, with no difference between the control groups (P>0.05; Fig. 4D).

## Discussion

Numerous studies have demonstrated that adiponectin is an important antiatherogenic adipocytokine that inhibits insulin resistance, inflammation and oxidative stress (21–25). In the present study, several important observations were noted. Firstly, adiponectin overexpression in atherosclerotic ApoE^−/−^ mice significantly reduced the size and area of carotid atherosclerotic lesions and positively changed the formation of the lesions. Secondly, it was identified that inhibiting superoxide production, preserving NO from destruction and blocking the formation of toxic ONOO^−^ are major mechanisms by which adiponectin exerts its antiatherosclerotic and vascular protective effects. Finally, adiponectin exerted an inhibitory effect on iNOS expression. Therefore, adiponectin reduces atherosclerosis, at least in part by antioxidative and antinitrative mechanisms, and may therefore be useful in the treatment of this condition.

Adiponectin is secreted by adipose tissue and has a significant role in the development of cardiovascular diseases. The incidence of cardiovascular mortality is increased in patients with low plasma adiponectin compared with that with higher plasma adiponectin levels (26). Furthermore, low adiponectin levels are associated with endothelial dysfunction and inflammation (27–29). Adiponectin infiltrates rapidly into the subendothelial space of the vascular wall when the endothelial barrier of the arterial wall is injured by balloon angioplasty (30) Another study documented that the overexpression of adiponectin actually reduced atherosclerosis through attenuating endothelial inflammatory response and macrophage-to-foam cell transformation *in vivo* (9). A previous study by our group also reported that transfection of Ad-APN in carotid arteries decreased the size and formation of atherosclerotic lesions (18). However, whether adiponectin reduces atherosclerosis by inhibiting iNOS and the resulting oxidative/nitrative stress remains to be confirmed by future studies.

Evidence suggests that common risk factors for atherosclerosis increase the risk of free ROS production from endothelial cells and from smooth muscle and adventitial cells (31). Hypercholesterolemia, diabetes mellitus, arterial hypertension, smoking, age and nitrate intolerance increase the production of free ROS, which includes superoxide anion (O_2_^−^), hydroxyl radical (OH^−^) and ONOO^−^. Superoxide reacts with NO at a near diffusion-limited rate, which is three times faster than the reaction between superoxide and superoxide dismutase (32). This reaction causes the inactivation of NO, a cytoprotective and vasodilatory molecule, and results in the formation of ONOO^−^, a highly reactive and cytotoxic molecule (33). However, adiponectin may significantly reverse this course. The present study provided direct evidence that adiponectin reduced the enhanced superoxide production in the carotid tissue of ApoE^−/−^ mice. It was demonstrated that NO production was decreased in Ad-APN-treated mice. The content of ONOO^−^, the biradical reaction product of superoxide and NO, was detected by measurement of nitrotyrosine, a marker of *in vivo* ONOO^−^ production, and the nitrotyrosine content was reduced with adiponectin pretreatment. Therefore, adiponectin reduced atherosclerosis in ApoE^−/−^ mice, likely through reducing superoxide production and preserving NO destruction.

Oxidative/nitrative stress due to increased iNOS and superoxide production and subsequent cytotoxic ONOO^−^ production are early hallmarks of vascular injury in patients with atherosclerosis. The physiological or pharmacological concentrations of NO exert significant cardioprotective effects, whereas the reaction product of NO and ·O_2_^−^, ONOO^−^, is highly cytotoxic. Several lines of evidence indicated that adiponectin improves endothelial function by its antioxidative/antinitrative properties and by enhancing eNOS activity, blocking iNOS and NADPH oxidase expression and ONOO^−^ production (1,34–36). A previous study by our group on cultured adventitial fibroblasts also demonstrated that adiponectin inhibited iNOS activity and reduced excessive NO production (17). In the present study, it was identified that adiponectin downregulated iNOS expression in carotid tissues. Levels of iNOS expression were increased in the carotid artery of ApoE^−/−^ mice and reduced significantly with overexpression of adiponectin. Further studies that examine these mechanisms may reveal novel signaling pathways that regulate iNOS activity.

In conclusion, these results demonstrated that adiponectin, as a unique cytokine, reduced atherosclerosis and attenuates oxidative/nitrative stress by blocking iNOS, superoxide and ONOO^−^ production. The present study took a different approach and provided evidence that adiponectin expression significantly reduced atherosclerosis associated with oxidative/nitrative stress.
